# An updated evaluation of serum sHER2, CA15.3, and CEA levels as biomarkers for the response of patients with metastatic breast cancer to trastuzumab-based therapies

**DOI:** 10.1371/journal.pone.0227356

**Published:** 2020-01-07

**Authors:** Alexandre Perrier, Pierre-Yves Boelle, Yves Chrétien, Joseph Gligorov, Jean-Pierre Lotz, Didier Brault, Eva Comperat, Guillaume Lefèvre, Mathieu Boissan

**Affiliations:** 1 Laboratoire de Biochimie et Hormonologie, Hôpital Tenon, Groupe Hospitalier Est Parisien, Assistance Publique–Hôpitaux de Paris, Paris, France; 2 Sorbonne Université, INSERM, Institut Pierre Louis d’Epidémiologie et de Santé Publique (IPLESP), Assistance Publique–Hôpitaux de Paris, Hôpital Saint-Antoine, Paris, France; 3 Centre de Recherche Saint-Antoine, INSERM, Sorbonne Université, Paris, France; 4 Service d’Oncologie Médicale, Institut Universitaire de Cancérologie APHP–Sorbonne Université, Paris, France; 5 Department of Pathology, Hôpital Tenon, Groupe Hospitalier Est Parisien, Assistance Publique Hôpitaux de Paris, Paris, France; Fondazione IRCCS Istituto Nazionale dei Tumori, ITALY

## Abstract

**Background:**

The transmembrane receptor tyrosine kinase HER2 is overexpressed in approximately 15% of breast tumors and correlates with poor clinical prognosis. Several treatments that target HER2 are approved for treatment of HER2-positive metastatic breast cancer. The serum biomarkers most widely used to monitor anti-HER2 therapies in patients with HER2-positive metastatic breast cancer currently are CA15.3 and CEA. Nevertheless, their clinical utility in patients with breast cancer remains a subject of discussion and controversy; thus, additional markers may prove useful in monitoring the therapeutic responses of these patients. The extracellular domain of HER2 can be shed by proteolytic cleavage into the circulation and this shed form, sHER2, is reported to be augmented during metastasis of HER2-positive breast tumors. Here, we studied the clinical usefulness of sHER2, CA15.3, and CEA for monitoring treatment for breast cancer.

**Methods:**

We measured prospectively pretreatment and post-treatment serum levels (day 1, 30, 60 and 90) of these three biomarkers in 47 HER2-positive, metastatic breast cancer patients treated with trastuzumab in combination with paclitaxel. Evaluation of the disease was performed according to the Response Evaluation Criteria in Solid Tumor (RECIST) at day 90.

**Results:**

Patients with progressive disease at day 90 had smaller relative changes between day 1 and day 30 than those with complete, partial or stable responses at day 90: -9% versus -38% for sHER2 (P = 0.02), +23% versus -17% for CA15.3 (P = 0.005) and +29% versus -26% for CEA (P = 0.02). Patients with progressive disease at day 90 were less likely than the other patients to have a relative decrease of > 20% in their biomarker levels at day 30: 6% vs 33% for sHER2 (P = 0.03), 0% vs 27% for CA15.3 (P = 0.03), 4% vs 29% for CEA (P = 0.04). No patient with progressive disease at day 90 had > 20% reduction of the average combined biomarker levels at day 30 whereas 63% of the other patients had (P = 0.003). Moreover, when we analyzed a > 10% reduction of the average biomarker levels no patient with progressive disease at day 90 had a decrease > 10% at day 30 whereas 78% of other patients had (P<0.001, Se = 100%, Sp = 78%).

**Conclusion:**

We show that regular measurement of sHER2, CA15.3, and CEA levels is useful for predicting the therapeutic response and for monitoring HER2-targeted therapy in patients with HER2-positive metastatic breast cancer. The average decrease of the three biomarkers with a threshold of > 10% appears to be the best parameter to distinguish patients who go on to have progressive disease from those who will have a complete, partial or stable response.

## Introduction

Breast cancer is the most frequent cancer in women; over a million new cases are diagnosed per year worldwide and thus this is an important health issue [1]. The transmembrane receptor tyrosine kinase HER2 (human epidermal growth factor receptor 2) is overexpressed in approximately 15% of breast tumors [2], and this overexpression is linked to poor clinical prognosis and disease progression [3]. Determination of HER2 status has become a necessary step in breast cancer diagnosis that is important not only for the prognosis but also for the choice of therapy. HER2 protein expression is most commonly measured in routine practice by immunohistochemistry. HER2-positive breast cancers respond to anti-HER2 treatments, particularly to monoclonal antibodies such as trastuzumab, which have significantly improved the prognosis for patients with non-metastatic and metastatic disease [4, 5].

The serum markers used most widely to predict clinical response to trastuzumab-based anti-HER2 therapy (*i*.*e*. trastuzumab alone or associated with adjuvant chemotherapy, such as paclitaxel) in patients with HER2-positive metastatic breast cancer are cancer antigen 15.3 (CA15.3) and carcinoembryonic antigen (CEA). Among the various tools used to assess the efficacy of a new therapy, the serum tumor markers CEA and CA15.3 are still a subject of discussion and controversy [6, 7]. Several studies have suggested that elevated serum levels of CA15.3 and CEA at the time of diagnosis are significantly associated with tumor size, axillary node metastasis and advanced stage in breast cancer patients [6; 8–10]. Others have found that, in addition, breast cancer patients with elevated CA15.3 and CEA levels have a poorer prognosis than those with normal levels of these markers [9–12]. Also, some studies suggest that CEA and CA15.3 may be useful as biomarkers to predict the therapeutic response in advanced breast cancer patients [12–15]. Nevertheless, the American Society of Clinical Oncology (ASCO) guidelines do not recommend use of CEA and CA15.3 alone for monitoring the response of breast cancer to treatment; they do, however, consider that these biomarkers may be used as adjunct assessments in the choice of therapy for metastatic breast cancer [16]. ASCO also notes that CEA and CA15.3 levels should be interpreted with caution during the first 4–6 weeks of administration of a new therapy because spurious increases may occur [16]. This may be due, in part, to conflicting conclusions reached by different researchers, but also to the fact that the serum of breast cancer patients is often not positive for these biomarkers or that they are not sensitive enough to detect the disease [7, 17]. Thus, other markers may prove useful in monitoring the therapeutic response of breast cancer patients [18].

In contrast to CEA and CA15.3, sHER2 is poorly documented as a biomarker for breast cancer although it is promising [18]. During HER2-positive breast cancer progression, tumor cells shed the extracellular domain (ECD) of HER2 by proteolytic cleavage [19], and this shedding is reported to be augmented during disease recurrence and metastasis [20–23]. Accordingly, the p105 kDa ECD or soluble HER2 (sHER2) can be detected in the circulation. High sHER2 levels pretreatment are reported to be significantly associated with an aggressive clinico-pathological phenotype [24]. Patients with decreased levels after receiving trastuzumab (with or without adjuvant chemotherapy) were found to be more likely to have a higher response rate and longer disease-free survival [20, 25–31]. In several studies of metastatic breast cancer, no clear relationship was found between baseline sHER2 levels and tumor response to trastuzumab-based treatment [32–34], whereas other studies have found a relationship [20, 31]; thus, no definitive conclusions can be drawn. Currently, ASCO does not recommend using sHER2 as a biomarker for the response to trastuzumab-based treatment because the evidence is too weak. There is only limited and controversial information regarding the usefulness of sHER2 to predict benefit from trastuzumab-based treatment in metastatic breast cancer patients. The current lack of a clear conclusion or consensus about this may be due to the use of various assays (commercial or home-made tests), different cut-off values, heterogeneity of the tumors, small numbers of patients investigated, short follow-up periods, and differences in the threshold and/or variation considered clinically significant. Nevertheless, the combination of several tumor markers, such as CA15.3, CEA and sHER2, might enhance the sensitivity for detection of metastatic breast cancer [35–37].

In this study, we set out to investigate whether the use of sHER2, CA15.3, and CEA would allow us to predict and to evaluate the responses of metastatic breast cancer patients to trastuzumab-based therapy. The innovative aspect of this study is the combined analysis of these three biomarkers to enhance the sensitivity for prediction of progressive disease and to discriminate between patients with progressive disease and other patients.

## Material and methods

### Study design and patients

Forty-seven patients with HER2-positive (level 3+ by immunohistochemistry or 2+ by immunohistochemistry associated with positive fluorescence *in situ* hybridization) first line metastatic breast cancer were recruited for a prospective evaluation of CEA, CA15-3 and sHER2 on treatment response prediction. In this study, we included patients with metastases who had not previously been treated and patients with metastases who had previously received treatment for their primary tumors. All the patients had been treated with a combination of trastuzumab (4 mg/kg on week 1, followed by 2 mg/kg/week) and paclitaxel (175 mg/m^2^ every 3 weeks or 80 mg/m^2^/weekly, 6 weeks/8) until progression or unacceptable toxicity. Evaluation of the disease was performed according to the Response Evaluation Criteria in Solid Tumor (RECIST). Inclusion criteria for these patients (IC) were: first line metastatic breast cancer measurable according to RECIST or not measurable (bone metastases, isolated pleural effusion) (IC 1), performance index (WHO) ≤ 2 (IC 2), life expectancy ≥ 3 months (IC 3), overexpression of HER2 (level 3+ by immunohistochemistry or 2+ by immunohistochemistry associated with positive FISH) (IC4), normal heart function (IC 5), polymorphonuclear neutrophils ≥ 1.5x10^9^/L (IC6), platelets ≥ 100x10^9^/L (IC 7), bilirubin ≤ 1.25N (IC 8), transaminases ≤ 2.5N (5N in case of liver metastases) (IC 9), alkaline phosphatases ≤ 2.5N (5N in case of liver metastases) (IC10), contraception if of childbearing age (IC 11). Exclusion criteria (EC) were: death (EC 1), cardiac pathology with left ventricular ejection < 50% at baseline (EC 2), oxygen-dependent lung disease (EC 3), antecedent of any other cancer except *in situ* carcinoma of the cervix (EC 4), symptomatic brain metastases (EC 5), adjuvant herceptin treatment (EC 6), toxicity and allergy related to paclitaxel (EC 7), free interval ≤ 12 months since a neoadjuvant treatment involving a taxane (EC 8), possibility of being treated with anthracyclines (EC 9), no relapse or progressive recovery following last treatment with anthracyclines (EC 10), positive HIV serology (EC 11), another condition preventing follow-up of the patient (EC 12). Written informed consent was obtained from the participants. The study protocol was approved by the Comité Consultatif De Protection des Personnes Dans La Recherche Biomédicale (CCPPRB PARIS-COCHIN; approval number Am2761-9-1878). All patients were recruited prospectively in Tenon Hospital (Groupe Hospitalier Est Parisien, Assistance Publique–Hôpitaux de Paris, Paris, France) between 2001 and 2005.

### Measurement of serum CEA and CA15.3

Serum levels of CEA and CA15.3 were measured by a chemiluminescent micro-particle immunoassay on ARCHITECT ci 8200 (ABBOTT^®^). The CA 15–3 assay values were defined by using the 115D8 and DF3 monoclonal antibodies supplied by ABBOTT^®^ [38–40]. Monoclonal antibody 115D8 raised against human milk-fat globule membranes, and monoclonal antibody DF3 raised against a membrane enriched fraction of metastatic human breast carcinoma, react with epitopes expressed by a family of high molecular weight glycoproteins designated as polymorphic epithelial mucins (PEMs) [41–44]. The anti-CEA assay used antibodies raised in guinea pig and goat and conjugated to peroxidase supplied by ABBOTT^®^. Both anti-CEA antibodies were raised against CEA purified from a colon cancer in tissue culture [41]. The approved threshold values were ≥ 30 U/mL for CA15.3, and ≥ 5 ng/mL for CEA. We collected blood samples for these two biomarkers at day 1, 30, 60 and 90.

### Measurement of serum extracellular domain of HER2

The ADVIA^®^ Centaur HER2 assay (SIEMENS^®^) is a fully automated, two-site sandwich immunoassay using direct chemiluminescent technology [45–47]. The Lite Reagent is composed of the monoclonal mouse antibody TA-1 labeled with acridinium ester. The Fluorescein Conjugate Reagent is composed of the monoclonal mouse antibody NB-3 labeled with fluorescein. These two monoclonal antibodies are specific for unique epitopes on the ECD of HER2. The solid phase is composed of purified anti-fluorescein monoclonal mouse capture antibody, which is covalently coupled to paramagnetic particles. The sample is incubated with Fluorescein Conjugate Reagent and Lite Reagent simultaneously for 5.5 minutes. After this incubation, the solid phase is added, and the mixture is incubated for an additional 2.75 minutes. After this final incubation, the immuno-complex formed is washed with water prior to initiation of the chemiluminescent reaction. The approved threshold value was ≥ 15 ng/mL. We collected blood samples for this biomarker at day 1, 30, 60 and 90.

### Immunohistochemistry

Breast tumor samples were fixed in formalin and embedded in paraffin blocks. Four-micrometer sections were cut then deparaffinized and rehydrated. Immunohistochemistry was performed by using a rabbit monoclonal antibody (clone 4B5; Ventana/Roche^®^) as the primary antibody and the modified streptavidin–biotin peroxidase method with diaminobenzidine as a chromogen. HER2 status was assessed only on primary tumors by performing immunohistochemistry. The IHC results identify three HER2 scores: 0 and 1+ scores with 10% cells with low and incomplete intensity labeling; score 2+ with at least 10% labeled cells with low or moderate but complete intensity labeling; score 3+ with more than 30% labeled cells with a strong and complete intensity labeling. 2+ scores are also referred to as ‘equivocal cases’ and include cases with heterogeneous HER2 overexpression. Only, the carcinomas with a 2+ score were analyzed for HER2 gene status by *in situ* hybridization. Positive controls with breast tumors constantly overexpressing HER2 and negative controls without antibody application were systematically performed.

### Statistical analyses

The changes in biomarker levels over time were analyzed statistically by applying the Wilcoxon signed rank and McNemar’s test (for changes in biomarker levels in the same individual) or the Mann–Whitney–Wilcoxon test and Fisher's exact test (to compare changes in biomarker levels of patients with progressive disease *versus* those of patients with a complete, partial, or stable response). We also performed diagnostic statistics. For all tests, a P value <0.05 was considered as significant. Statistical analyses and figures were performed with the R^®^ software version 3.5.2.

## Results

**Fig 1:**
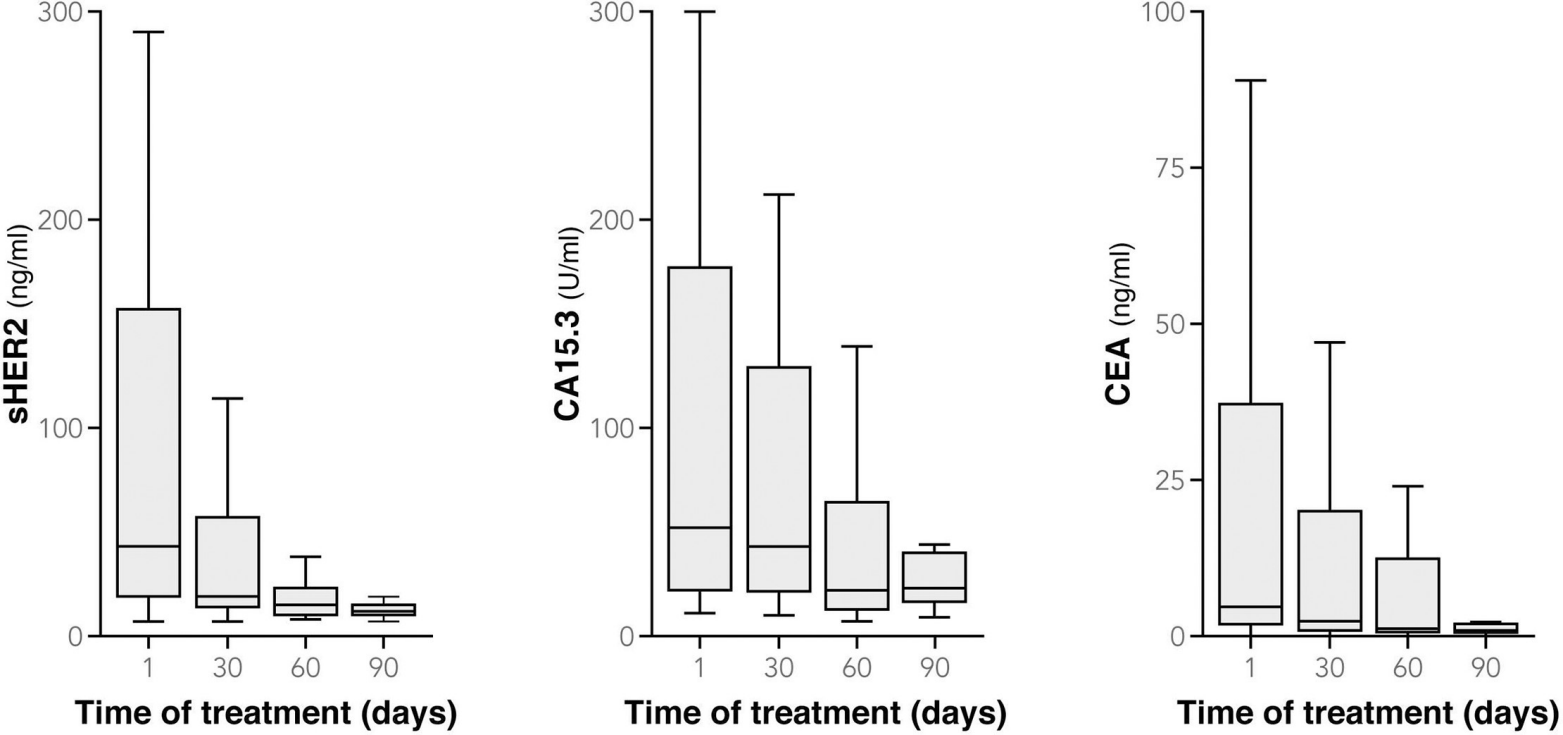
Boxplots of sHER2, CA15.3 and CEA levels at day 1, 30, 60 and day 90 (extreme values of biomarker levels are not represented but are included in the statistical analysis).

Patient and tumor characteristics at the diagnosis are detailed in Table 1. On day 1, the median value of sHER2 in our patient sample was 34 [range 7–4180] ng/mL, CA15.3 was 52 [range 11–2850] U/mL, and CEA was 4.6 [range 0–910] ng/mL (Fig 1); 87% of patients (41/47) had sHER2 levels ≥ 15 ng/mL, 64% (28/44) had CA15.3 ≥ 30 U/mL and 48% (21/44) had CEA ≥ 5 ng/mL (Table 2). We compared the three biomarker levels (Table 3) and observed that at day 1 patients with HER2-postive metastatic breast cancer were more often above the threshold for sHER2 than they were for CA15.3 (P = 0.01) and CEA (P<0.001), whereas no statistically significant differences were seen between the proportion of patients with CA15.3 and CEA levels above the threshold (P = 0.19). The therapeutic response was evaluated at day 90 according to the Response Evaluation Criteria in Solid Tumors (RECIST). At this time, 27 patients had partially (24 patients) or completely responded to treatment (3 patients), 13 had stable disease and 7 had progressive disease.

**Table 1:** Patient and tumor characteristics.

Characteristics of the 47 breast metastatic breast cancer patients	
Number of patients	47
Age (years)	
Average (range)	56 (26–75)
Median (SD)	58 (9.96)
<45 years (%)	7 (14.9%)
⩾ 45 to <55 years (%)	10 (21.3%)
⩾ 55 years (%)	30 (63.8%)
Menopause status	
Number of menopausal patients (%)	40 (85.1%)
Average (range)	51 (42–59)
Median (SD)	51 (3.64)
Predictive factors	
Progesterone Receptor + (%)	15 (31.9%)
Estrogen Receptor + (%)	19 (40.4%)
HER2+ (%)	47 (100%)
HER2+ 3+ (%)	42 (89.4%)
HER2+ 2+ ISH+ (%)	5 (10.6%)
Hormone replacement therapy (%)	14 (29.8%)
Metastasis	
Average number of sites per patients (range)	1.93 (1–4)
Number of patients with liver metastasis (%)	22 (46.8%)
Number of patients with bone metastasis (%)	17 (36.2%)
Number of patients with lung metastasis (%)	11 (23.4%)
Number of patients with cutaneous metastasis (%)	6 (12.8%)
Number of patients with other(s) location(s) (%)	10 (21.3%)
Therapeutic response at day 90 (according to RECIST)	
Complete response (%)	3 (6.4%)
Partial response (%)	24 (51.1%)
Stable (%)	13 (27.7%)
Progression (%)	7 (14.9%)

**Table 2:** Statistical analysis of changes in individual biomarker levels (sHER2, CA15.3 and CEA) over time (Day 1, 30 and 90) and according to the therapeutic response to treatment of HER2-positive metastatic breast cancer patients with a combination of trastuzumab and paclitaxel.

Biomarker	sHER2		CA15.3		CEA	
Threshold	≥ 15 ng/mL		≥ 30 U/mL		≥ 5 ng/mL	
**Pre-treatment (Day 1)**						
Median value [Range]	34 ng/mL [7–4180]		52 U/mL [11–2850]		4.6 ng/mL [0–910]	
% patients with biomarker levels above the threshold	87% (41/47)		64% (28/44)		48% (21/44)	
**Post-treatment (Day 30)**						
Overall change of biomarker levels between day 1 and day 30	-34% (± 5%*)	P<0.001^a^	-11% (± 6%*)	P = 0.02^a^	-17% (± 11%*)	P = 0.03^a^
% patients with biomarker levels above the threshold at day 1 decreasing below the threshold at day 30	22% (9/41)		0% (0/28)		24% (5/21)	
% change in biomarker levels of progressors *vs* those of patients with a complete, partial, or stable response between day 1 and day 30	-9% (±7%*) *vs* -38% (±6%*)	P = 0.02^b^	+23% (±13%*) *vs* -17% (±7%*)	P = 0.005^b^	+29% (±20%*) *vs* -26% (±12%*)	P = 0.02^b^
% progressors with biomarker levels above the threshold at day 30 *vs* patients with a complete, partial or stable response	86% (6/7) *vs* 65% (26/40)	P = 0.40^c^	71% (5/7) *vs* 62% (23/37)	P = 1.00^c^	71% (5/7) *vs* 32% (12/37)	P = 0.09^c^
% patients with > 20% reduction of biomarker levels	68% (32/47)		41% (18/44)		52% (23/44)	
% progressors among patients with > 20% reduction of biomarker levels *vs* % progressors among patients with < 20% reduction of biomarker levels	6% (2/32) *vs* 33% (5/15)	P = 0.03^c^	0% (0/18) *vs* 27% (7/26)	P = 0.03^c^	4% (1/23) *vs* 29% (6/21)	P = 0.04^c^
% patients with > 10% reduction of biomarker levels	72% (34/47)		61% (27/44)		59% (26/44)	
% progressors in patients with > 10% reduction of biomarker levels *vs* % progressors in patients with < 10% reduction of biomarker levels	6% (2/34) *vs* 38% (5/13)	P = 0.01^c^	4% (1/27) *vs* 35% (6/17)	P = 0.01^c^	8% (2/26) *vs* 28% (5/18)	P = 0.10^c^
**Post-treatment (Day 90)**						
Overall change of biomarker levels between day 1 and day 90	-51% (±8%*)	P<0.001^a^	-22% (±9%*)	P = 0.01^a^	-35% (±13%*)	P = 0.001^a^
% patients with biomarker levels above the threshold (P-value for comparison to day 1)	35% (12/34)	P<0.001^d^	46% (16/35)	P = 0.07^d^	20% (7/35)	P = 0.03^d^
% change in biomarker levels of progressors *vs* those of patients with a complete, partial, or stable response between day 1 and day 90	-8% (±32%*) *vs* -57% (±8%*)	P = 0.08^b^	+36% (±29%*) vs -30% (±9%*)	P = 0.04^b^	+32% (±54%*) *vs* -44% (±13%*)	P = 0.04^b^

a: Wilcoxon signed rank test

b: Mann–Whitney–Wilcoxon test

c: Fisher's exact test

d: McNemar’s test

*: Standard error of the mean

**Table 3:** Comparison of biomarker levels (sHER2, CA15.3 and CEA) over time (Day 1, 30 and 90) and according to the therapeutic response to treatment of HER2-positive metastatic breast cancer patients with a combination of trastuzumab and paclitaxel.

Biomarkers compared	sHER2 *vs* CA15.3		sHER2 *vs* CEA			CA15.3 *vs* CEA	
**Pre-treatment (Day 1)**							
% patients with biomarker levels above the threshold	87% (41/47) *vs* 64% (28/44)	P = 0.01^b^	87% (41/47) *vs* 48% (21/44)		P<0.001^b^	64% (28/44) *vs* 48% (21/44)	P = 0.19^b^
**Post-treatment (Day 30)**							
Overall change of biomarker levels between day 1 and day 30	-34% (± 5%*) *vs* -11% (± 6%*)	P<0.001^a^	-34% (± 5%*) *vs* -17% (± 11%*)	P = 0.23^a^		-11% (± 6%*) *vs* -17% (± 11%*)	P = 0.20^a^
% patients with biomarker levels above the threshold at day 1 that decreased below the threshold at day 30	22% (9/41) *vs* 0% (0/28)	P = 0.009^b^	22% (9/41) *vs* 24% (5/21)	P = 1.00^b^		0% (0/28) *vs* 24% (5/21)	P = 0.01^b^
% change in biomarker levels of progressors between day 1 and day 30	-9% (±7%*) *vs* +23% (±13%*)	P = 0.05^a^	-9% (±7%*) *vs* +29% (±20%*)	P = 0.08^a^		+23% (±13%*) *vs* +29% (±20%*)	P = 0.81^a^
% change in biomarker levels of patients with a complete, partial, or stable response between day 1 and day 30	-38% (±6%*) *vs* -17% (±7%*)	P<0.001^a^	-38% (±6%*) *vs* -26% (±12%*)	P = 0.62^a^		-17% (±7%*) *vs* -26% (±12%*)	P = 0.11^a^
% patients with > 20% reduction of biomarker levels	68% (32/47) *vs* 41% (18/44)	P = 0.01^b^	68% (32/47) *vs* 52% (23/44)	P = 0.14^b^		41% (18/44) *vs* 52% (23/44)	P = 0.39^b^
% patients with > 10% reduction of biomarker levels	72% (34/47) *vs* 61% (27/44)	P = 0.37^b^	72% (34/47) *vs* 59% (26/44)	P = 0.19^b^		61% (27/44) *vs* 59% (26/44)	P = 1.00^b^
**Post-treatment (Day 90)**							
Overall change of biomarker levels between day 1 and day 90	-51% (±8%*) *vs* -22% (±9%*)	P = 0.002^a^	-51% (±8%*) *vs* -35% (±13%*)	P = 0.19^a^		-22% (±9%*) *vs* -35% (±13%*)	P = 0.14^a^
% patients with biomarker levels above the threshold	35% (12/34) *vs* 46% (16/35)	P = 0.46^b^	35% (12/34) *vs* 20% (7/35)	P = 0.19^b^		46% (16/35) *vs* 20% (7/35)	P = 0.04^a^
% change in biomarker levels of progressors between day 1 and day 90	-8% (±32%*) *vs* +36% (±29%*)	P = 0.63^a^	-8% (±32%*) *vs* +32% (±54%*)	P = 0.38^a^		+36% (±29%*) *vs* +32% (±54%*)	P = 1.00^a^
% change in biomarker levels of patients with a complete, partial, or stable response between day 1 and day 90	-57% (±8%*) *vs* -30% (±9%*)	P = 1.00^a^	-57% (±8%*) *vs* -44% (±13%*)	P = 0.38^a^		-30% (±9%*) *vs* -44% (±13%*)	P = 0.09^a^
% patients with > 20% reduction of biomarker levels	85% (29/34) *vs* 54% (19/35)	P = 0.008^b^	85% (29/34) *vs* 66% (23/35)	P = 0.09^b^		54% (19/35) *vs* 66% (23/35)	P = 0.46^a^
% patients with > 10% reduction of biomarker levels	85% (29/34) *vs* 63% (22/35)	P = 0.05^b^	85% (29/34) *vs* 66% (23/35)	P = 0.09^b^		63% (22/35) *vs* 66% (23/35)	P = 1.00^a^

a: Wilcoxon signed rank test

b: Fisher's exact test

*: Standard error of the mean

**Fig 2:**
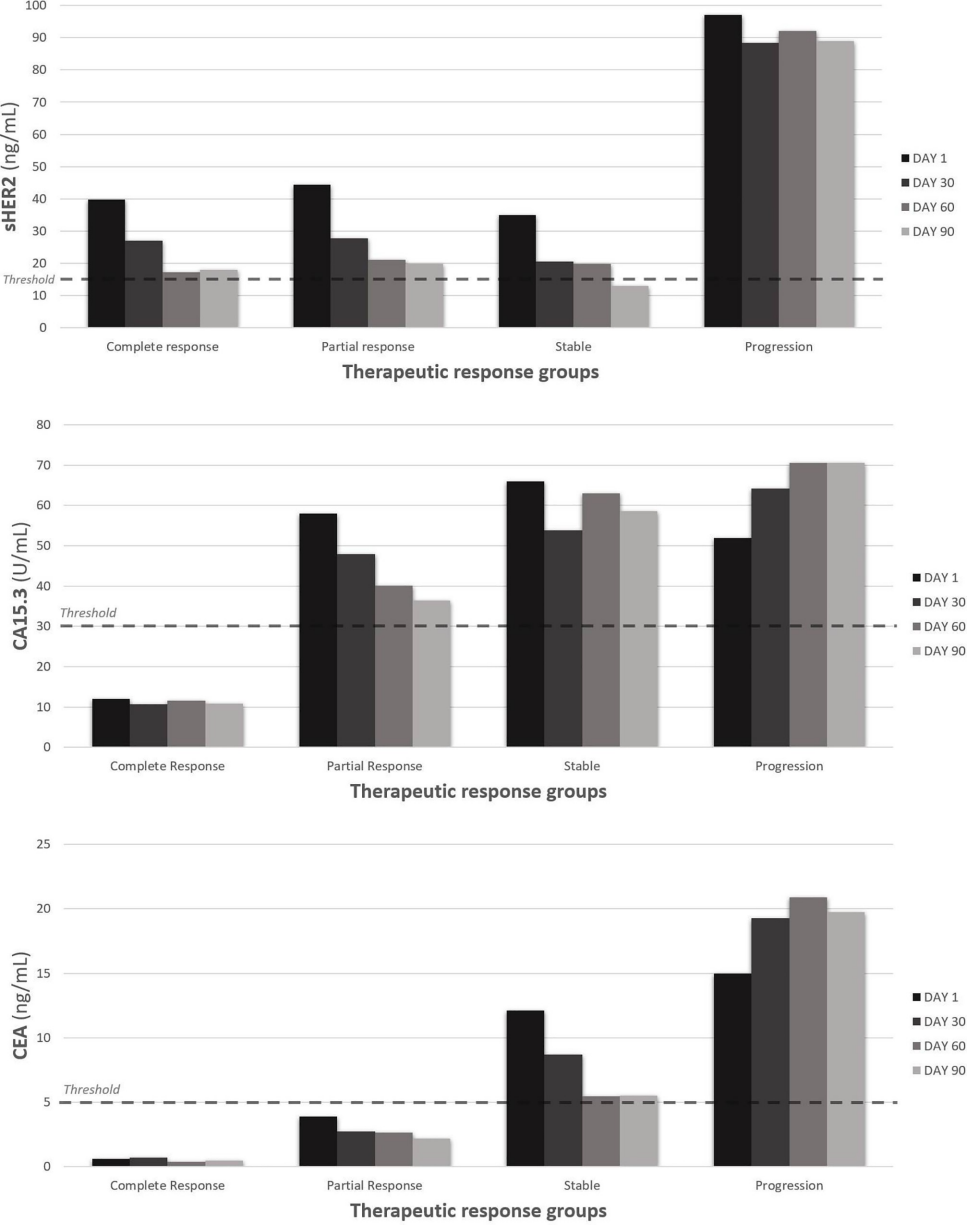
Kinetics of biomarker levels (sHER2, CA 15.3, CEA) over time (at day 1, 30, 60 and 90) in the different therapeutic response groups (patients with a complete, partial or stable response and patients with progressive disease), based on RECIST criteria at day 90.

We evaluated the kinetics of change in the three biomarker levels (sHER2, CA15.3 and CEA) at day 1, 30, 60 and 90 in the different therapeutic response groups (Fig 2) Between day 1 and day 30 of treatment, we observed an average decrease of 34% in serum levels of sHER2 (P<0.001), 11% in CA15.3 (P = 0.02) and 17% in CEA (P = 0.03) (Fig 1; Table 2). sHER2 levels decreased more than those of CA15.3 (P<0.001), but not more than CEA (P = 0.23), and no differences were found between CA15.3 and CEA (P = 0.20). All patients with an initial serum biomarker level below the threshold remained so on day 30 (except one patient with progressive disease whose CEA levels rose above the threshold); those with an initial serum biomarker level above the threshold had decreased below the threshold on day 30 in 22% (9/41) of the cases for sHER2, 0% (0/28) for CA15.3 and 24% (5/21) for CEA. The results were significantly different when sHER2 was compared with CA15.3 (P = 0.009) and when CA15.3 was compared with CEA (P = 0.01), but not when sHER2 was compared with CEA (P = 1.00).

**Fig 3:**
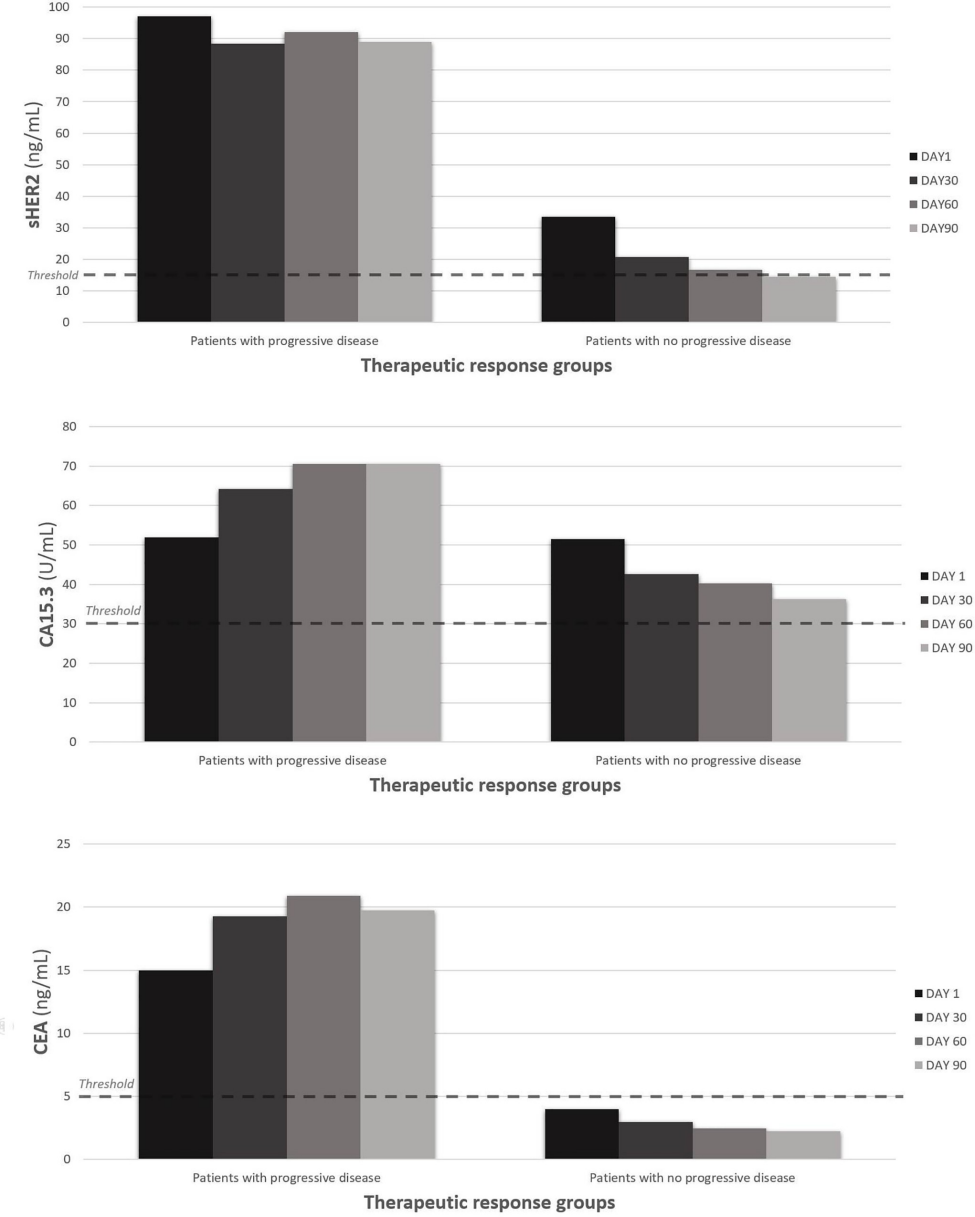
Kinetics of biomarker levels (sHER2, CA 15.3, CEA) over time (at day 1, 30, 60 and 90) in patients with progressive disease versus patients with no progressive disease (patients with a complete, partial or stable response), based on RECIST criteria at day 90.

Patients with progressive disease at day 90 had smaller relative changes between day 1 and day 30 than those with complete, partial or stable responses at day 90: -9% versus -38% for sHER2 (P = 0.02), +23% versus -17% for CA15.3 (P = 0.005) and +29% versus -26% for CEA (P = 0.02) (Fig 3). We observed a decrease of biomarker levels in patients with complete, partial or stable responses for sHER2, CA15.3 and CEA (Fig 2). Mean values of patients with complete response were below threshold from the first day until day 90 for CA15.3 and CEA (Fig 2). This was also the case for patients with partial response for CEA (Fig 2). The sHER2 levels in patients with a complete, partial or stable response decreased more than CA15.3 levels (P<0.001) but not significantly more than CEA (P = 0.62). In patients with progressive disease, we also observed a tendency of sHER2 to decrease more than CA15.3 (P = 0.05). We saw no significant difference between patients with progressive disease at day 90 and the other patients when we analyzed serum biomarker levels above the threshold at day 30: 86% (6/7) versus 65% (26/40) for HER2 (P = 0.40), 71% (5/7) versus 32% (12/37) for CEA (P = 0.09) and 71% (5/7) versus 62% (23/37) for CA15.3 (P = 1.00). The small number of patients with progressive disease limits the statistical power of these comparisons.

Patients with progressive disease at day 90 were less likely than the other patients to have a relative decrease of > 20% in their biomarker levels at day 30. For sHER2 (n = 47), 68% (32/47) of all patients’ serum levels decreased by > 20%, among which only 6% (2/32) had progressive disease; among the 15 patients whose serum levels decreased by < 20%, by contrast, 33% (5/15) had progressive disease (P = 0.03), (Se = 71%, Sp = 75%, Table 4). For CA15.3 (n = 44), 41% (18/44) of all patients’ serum levels decreased by > 20%, among which none (0/18) had progressive disease; among the 26 patients whose serum levels decreased by < 20%, 27% (7/26) had progressive disease (P = 0.03), (Se = 100%, Sp = 49%). For CEA (n = 44), 52% (23/44) of all patients’ serum levels decreased by > 20%, among which 4% (1/23) had progressive disease; among the 21 patients whose serum levels decreased by < 20%, 29% (6/21) had progressive disease (P = 0.04), (Se = 86%, Sp = 59%). We obtained similar results when we analyzed the patients that had a relative decrease of > 10% in their biomarker levels at day 30. We found that more patients had a reduction of > 20% when sHER2 was used as a biomarker instead of CA15.3 (P = 0.01) and we observed no differences between sHER2 and CEA (P = 0.14) and between CA15.3 and CEA (P = 0.39). When we applied a threshold of > 10% reduction, no differences were noticed. Despite the low number of patients with progressive disease, we observed that they had a significantly smaller decrease in the three biomarker levels than had the other patients at day 30.

**Table 4:** Diagnostic statistics of the three biomarker levels (sHER2, CA15.3 and CEA) for detecting progression.

Parameter tested: progressive disease	sHER2	CA15-3	ACE
Post-treatment Day 30			
Test: Biomarker levels above the threshold			
Sensitivity	86%	71%	71%
Specificity	35%	38%	68%
Positive predictive value	19%	18%	29%
Negative predictive value	93%	88%	88%
Accuracy	43%	40%	64%
Test: reduction of biomarker levels (threshold 20%)			
Sensitivity	71%	100%	86%
Specificity	75%	49%	59%
Positive predictive value	33%	27%	29%
Negative predictive value	94%	100%	96%
Accuracy	74%	57%	64%
Test: reduction of biomarker levels (threshold 10%)			
Sensitivity	71%	86%	71%
Specificity	80%	74%	65%
Positive predictive value	38%	35%	28%
Negative predictive value	94%	96%	92%
Accuracy	79%	73%	66%

We calculated the relative change in biomarker levels between day 1 and day 90 in all patients. The mean serum level of sHER2 in all patients decreased by 51% (P<0.001), that of CA15.3 by 22% (P = 0.01) and of CEA by 35% (P = 0.001) (Fig 1; Table 2). Levels of sHER2 decreased more than CA15.3 (P = 0.002) but not in comparison with CEA (P = 0.19), and no differences were found between CA15.3 and CEA (P = 0.14). At day 90, 35% of patients had levels of sHER2 ≥ 15 ng/mL, 46% had levels of CA15.3 ≥ 30 U/mL and 20% had levels of CEA ≥ 5 ng/mL, compared with 87% (P<0.001), 64% (P = 0.07) and 48% (P = 0.03) respectively, at day 1. More patients had CA15.3 levels above the threshold than had CEA levels above the threshold (P = 0.04), whereas no differences were seen between the other biomarkers. Patients with progressive disease had much smaller changes than the other patients between day 1 and day 90: -8% versus -57% for sHER2 (P = 0.08), +36% versus -30% for CA15.3 (P = 0.04) and +32% versus -44% for CEA (P = 0.04) (Fig 3). Similar biomarker level changes were seen in patients with progressive disease when we compared sHER2 to CA15.3 (P = 0.63), sHER2 to CEA (P = 0.38), and CA15.3 to CEA (P = 1.00) between day 1 and day 90. That was also the case for patients with a complete, partial or stable response: comparing sHER2 to CA15.3 (P = 1.00), sHER2 to CEA (P = 0.38), and CA15.3 to CEA (P = 0.09). Mean values of patients with no progressive disease have decreased below threshold at day 90 for sHER2 and CEA, not for CA15.3 (Fig 3). Considering all the patients, however, more had a > 20% reduction in sHER2 levels with between day 1 and day 90 than had a > 20% reduction in CA15.3 levels (P = 0.008); this held true also when a > 10% reduction in sHER2 and CA15.3 levels was analyzed (P = 0.05). In light of these data from day 30 and day 90 of treatment, we suggest that sHER2, and to a lesser degree CEA, levels are a little more sensitive to clinical changes than are CA15.3 levels.

**Fig 4:**
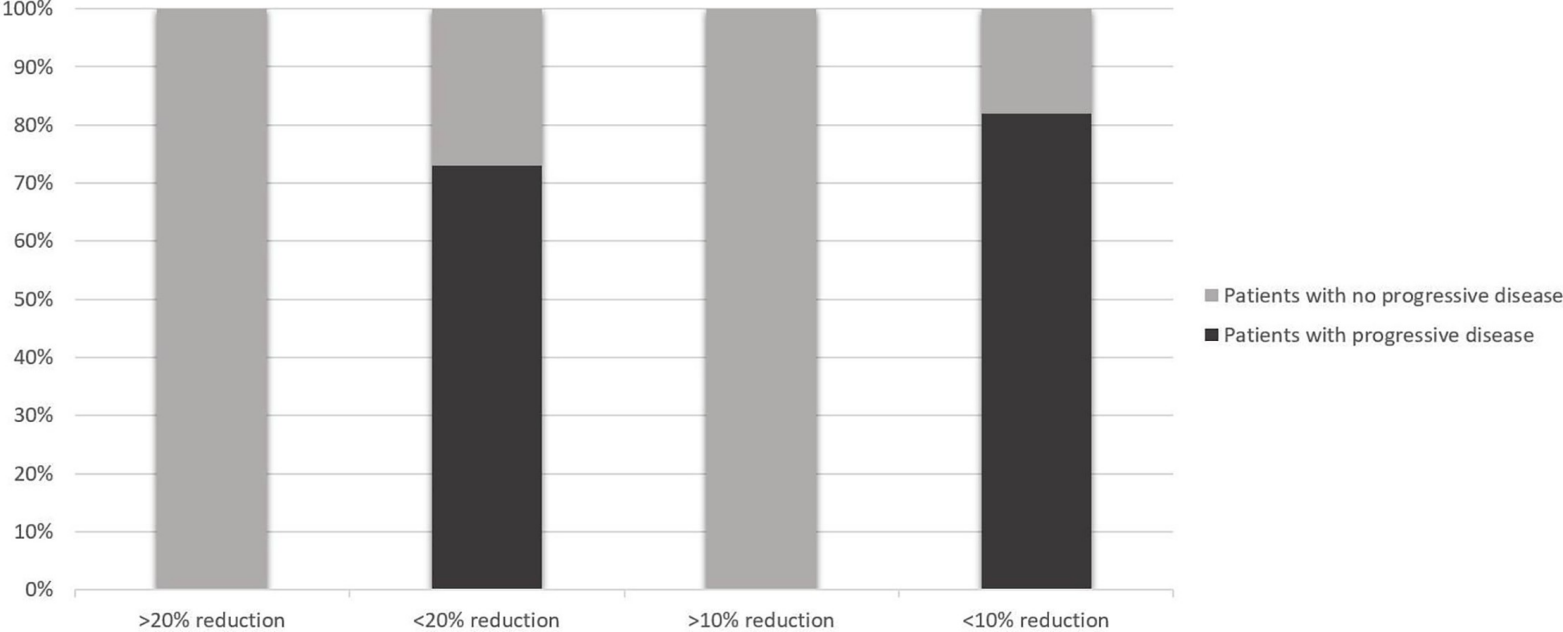
The proportion of patients who at day 30 have a > or < 20% reduction in their average biomarker level, or a > or < 10% reduction in their average biomarker level and who go on to have progressive disease (according to RECIST criteria) at day 90 (black shading) or to have no progressive disease (i.e. a complete, partial or stable response; grey shading).

Finally, we investigated whether a combination of the three tumor biomarkers together (CA15.3, CEA and sHER2) might enhance the sensitivity of detection of progressive disease (Table 5 and Table 6). Seventy-five percent (33/44) of all patients were above the threshold for at least two of the three biomarkers at day 1. The average change in the three biomarker levels in all patients between day 1 and day 30 was -22% (P<0.001). Patients with progressive disease at day 90 had a smaller relative average change in the three biomarkers between day 1 and day 30 than had the other patients: +14% versus -27% (P<0.001). Eighty-six percent (6/7) of patients with progressive disease had at least two of the three biomarker levels above the threshold at day 30 compared to 57% (21/37) of the other patients (P = 0.22). Fifty-three percent (25/47) of all patients had a > 20% reduction of the average biomarker levels at day 30 and 66% (31/47) had a reduction of > 10%. Combination of the three biomarkers appears to permit better discrimination between patients with progressive disease and other patients. For instance, no patient (0/7) with progressive disease at day 90 had > 20% reduction of the average biomarker levels at day 30 whereas 63% (25/40) of the other patients had (P = 0.003), (Se = 100%, Sp = 63%, PPV = 32%, NPV = 100%, Accuracy = 68%) (Fig 4 and Table 6). Moreover, when we analyzed a > 10% reduction of the average biomarker levels: no patient (0/7) with progressive disease at day 90 had a decrease > 10% at day 30 whereas 78% (31/40) of other patients had (P<0.001) (Fig 4). We observed with this threshold an increase of specificity and positive predictive value without sensitivity and negative predictive value decreasing (Se = 100%, Sp = 78%, PPV = 44%, NPV = 100%, Accuracy = 81%) (Table 6). When we analyzed a reduction of > 20% in levels of at least two of the three biomarkers, we also found a significant difference between patients with progressive disease and others: 14% (1/7) versus 65% (25/40) (P = 0.03). Similar results were found by analyzing a reduction of > 10% in levels of at least two of the three biomarkers: 29% (2/7) of patients with progressive disease versus 76% (28/37) of other patients (P = 0.03).

**Table 5:** Statistical analysis of changes in combined biomarker levels (sHER2, CA15.3 and CEA) over time (Day 1, 30 and 90) and according to the therapeutic response to treatment of HER2-positive metastatic breast cancer patients with a combination of trastuzumab and paclitaxel.

**Pre-treatment (Day 1)**		
% patients with at least two of the three biomarker levels above the threshold	75% (33/44)	
**Post-treatment (Day 30)**		
Mean change in the three biomarker levels between day 1 and day 30	-22% (± 5%*)	P<0.001^a^
Mean change in the three biomarker levels in progressors between day 1 and day 30 *vs* that in patients with a complete, partial, or stable response	+14% (±12%*) *vs* -27% (±4%*)	P<0.001^b^
% progressors with at least two of the three biomarker levels above the threshold at day 30 *vs* the % of patients with a complete, partial, or stable response with at least two of the three biomarker levels above the threshold at day 30	86% (6/7) *vs* 57% (21/37)	P = 0.22^c^
% patients with > 20% reduction of the average biomarker levels	53% (25/47)	
% progressors with > 20% reduction of the average biomarker levels *vs* % of patients with a complete, partial, or stable response with > 20% reduction of the average biomarker levels	0% (0/7) *vs* 63% (25/40)	P = 0.003^c^
% progressors with > 20% reduction in at least two of the three biomarker levels *vs* % of patients with a complete, partial, or stable response with > 20% reduction in at least two of the three biomarker levels	14% (1/7) *vs* 65% (24/37)	P = 0.03^c^
% patients with > 10% reduction of the average biomarker levels	66% (31/47)	
% progressors with > 10% reduction in the average biomarker levels *vs* % of patients with a complete, partial, or stable response with > 10% reduction in the average biomarker levels	0% (0/7) *vs* 78% (31/40)	P<0.001^c^
% progressors with > 10% reduction in at least two of the three biomarker levels *vs* % of patients with a complete, partial, or stable response with > 10% reduction in at least two of the three biomarker levels	29% (2/7) *vs* 76% (28/37)	P = 0.03^c^
**Post-treatment (Day 90)**		
Mean change in the three biomarker levels between day 1 and day 90	-36% (±6%*)	P<0.001^a^
% patients with at least two of the three biomarker levels above the threshold (P-value for comparison to day 1)	32% (11/34)	P = 0.001^d^
Mean change in the three biomarker levels in progressors between day 1 and day 90 *vs* patients with a complete, partial, or stable response	+20% (±22%*) *vs* -44% (±6%*)	P = 0.04^b^

a: Wilcoxon signed rank test

b: Mann–Whitney–Wilcoxon test

c: Fisher's exact test

d: McNemar’s test

*: Standard error of the mean

**Table 6:** Diagnostic statistics of combined biomarker levels (sHER2, CA15.3 and CEA).

Parameter tested: progression	Combined biomarkers: sHER2, CA15.3, CEA
Post-treatment Day 30	
Test: at least two of the three biomarker levels above the threshold	
Sensitivity	86%
Specificity	43%
Positive predictive value	22%
Negative predictive value	94%
Accuracy	47%
Test: reduction of the average biomarker levels (threshold 20%)	
Sensitivity	100%
Specificity	63%
Positive predictive value	32%
Negative predictive value	100%
Accuracy	68%
Test: reduction of the average biomarker levels (threshold 10%)	
Sensitivity	100%
Specificity	78%
Positive predictive value	44%
Negative predictive value	100%
Accuracy	81%
Test: reduction in at least two of the three biomarker levels (threshold 20%)	
Sensitivity	86%
Specificity	65%
Positive predictive value	32%
Negative predictive value	96%
Accuracy	68%
Test: reduction in at least two of the three biomarker levels (threshold 10%)	
Sensitivity	71%
Specificity	76%
Positive predictive value	36%
Negative predictive value	93%
Accuracy	75%

Considering all the data above, the best parameter for use in clinical practice seems to be the average decrease of the three biomarkers. At day 90, the average change of the three biomarker levels was -36% (P<0.001). At day 90, 32% (11/34) of patients had at least two of the three biomarker levels above the threshold compared with 75% (33/44) at day 1 (P = 0.001). Between day 1 and day 90, patients with progressive disease had smaller relative changes in the average biomarker levels than had the other patients: +20% versus -44% (P = 0.04).

## Discussion

In this prospective study, we aimed to find a combination of biomarkers that would identify patients who are likely to respond to trastuzumab-based therapies. In terms of individual biomarkers, we find that a large decrease (either of >10% or of >20%) in any of the three biomarkers we evaluated–sHER2, CEA and CA15.3 –in the first month of treatment is a strong indicator against disease progression in metastatic breast cancer patients treated with a combination of trastuzumab and paclitaxel. Moreover, levels of sHER2 and CEA seem to be generally more sensitive indicators of clinical change than are levels of CA15.3. One of the strengths of this study, however, is its unusual use of a combined analysis of three biomarkers. We find that this combination can enhance the ability to discriminate between patients with progressive disease and patients with a complete, partial or stable response. The best parameter seems to be the average decrease of the three biomarkers. Analyzing the kinetics of change in the levels of the three biomarkers appears to be more useful than analyzing actual biomarker levels with respect to a fixed threshold level when distinguishing between patients with progressive disease and other patients. By applying a threshold of >10% or >20% reduction of average biomarker levels, we can very robustly identify patients who are likely to respond to treatment.

When we performed diagnostic statistics based on detection of progressive disease, we obtained a sensitivity of 100% and a negative predictive value of 100% with a threshold of > 10% or > 20% reduction of the average biomarker levels. There were no false negatives (i.e. patients with progressive disease but a decrease greater than the threshold of > 10% or > 20%). The very high negative predictive value is particularly useful in clinical practice: patients with a decrease of > 10% or > 20% are probably not going to have progressive disease. The specificity and especially the positive predictive value were low, however. Thus, a patient with < 10% or < 20% decrease of the three combined biomarker levels is not necessarily a patient with progressive disease. The threshold of > 10% average decrease of biomarker levels seems to be more useful than the threshold of > 20% to distinguish patients with progressive disease from other patients because the specificity and the positive predictive value are higher at the > 10% threshold than at the > 20% threshold and the sensitivity and the negative predictive value are also high; thus, there is a reduction of the number of false positives (i.e. patients with no progressive disease but a decrease of average biomarker levels < 10%).

The main limitation of our study is the small number of patients with progressive disease (7 patients in the cohort of 47), which limits the statistical significance of the observed average decrease of sHER2 levels in patients with progressive disease. Thus, we cannot draw strong conclusions from this study, but we can develop hypotheses for future validation. The difficulties involved in calculating changes in combined biomarker levels in clinical practice may prove an impediment to the application of the method. To overcome this problem, we plan to develop a free, online tool to facilitate the calculation. Another potential limitation of the study is that the therapeutic response was evaluated only at day 90. Thus, we cannot be certain that some patients who were classified as stable or as responders at day 90 did not have progressive disease at day 60 or day 30 or, vice versa, that some patients progressed tardily, so were stable or responders at day 30 or day 60 but were classified as having progressive disease at day 90. Despite these limitations, this study indicates that sHER2, CA15.3 and CEA may together be useful as an adjunct tool for predicting progressive disease in the first-line treatment of HER2-positive metastatic breast cancer patients. We need a validation group to determine whether the findings obtained are generally applicable. So, we plan to conduct a multi-center prospective study with a large cohort to confirm these results.

## Conclusion

We conclude that early measurement of sHER2, CA15.3, and CEA levels in serum can be informative about the eventual outcome for metastatic breast cancer patients treated with a combination of trastuzumab and paclitaxel: among those with a large decrease (> 10%) in biomarker levels in the first month of treatment, few patients go on to have progressive disease. Hence, serial measurements of sHER2, CA15.3, and CEA over the course of treatment may prove useful for monitoring HER2-targeted therapies and predicting disease progression. Combined analysis of the three biomarker levels allows a more accurate interpretation of biomarker kinetics and permits better discrimination between patients with progressive disease and patients with a complete, partial or stable response than does a simple analysis of any one of the biomarkers. Levels of sHER2 and CEA seem to be more sensitive indicators of clinical changes than are levels of CA15.3 in some situations. The average decrease of the three biomarkers with a threshold of > 10% appears to be the best parameter to distinguish patients with progressive disease from other patients. Although the sample size was small, this pilot study provides useful and encouraging information to design multi-center prospective studies that would evaluate further the prognostic value of measuring serum concentrations of sHER2, CA15.3, and CEA as markers of responses to trastuzumab-based treatments.

## Supporting information

S1 Dataset (XLSX)

## Abbreviations

ASCO: American Society of Clinical Oncology
CA15.3: cancer antigen 15.3
CEA: carcinoembryonic antigen
ECD: extracellular domain
ER: estrogen receptor
PR: progesterone receptor
sHER2: soluble human epidermal growth factor receptor 2.
